# Utilizing Albumin Value, HALP Score and LCR Value for Predicting Survival in Patients with Pancreatic Adenocarcinoma

**DOI:** 10.3390/medicina61040639

**Published:** 2025-04-01

**Authors:** Tufan Gumus, Veysel Umman, Bekir Cetin, Alper Uguz

**Affiliations:** 1 Department of General Surgery, Faculty of Medicine, Ege University, 35040 Izmir, Türkiye; 2 Department of General Surgery, Faculty of Medicine, Yeditepe University, 34755 Istanbul, Türkiye

**Keywords:** pancreatic adenocancer, albumin, hemoglobin, lymphocyte, platelet

## Abstract

*Background and Objectives*: This study aimed to determine whether albumin levels and the ratios of key biochemical markers, including the hemoglobin, albumin, lymphocyte, and platelet (HALP score) and lymphocyte/C-reactive protein ratio (LCR), can predict survival and recurrence in patients with pancreatic adenocarcinoma. *Materials and Methods*: A total of 87 patients who underwent surgery for pancreatic adenocarcinoma in our clinic between January 2017 and December 2021 were included. Preoperative albumin levels, HALP scores, and LCR values were calculated and analyzed to evaluate their predictive value for pathological findings in the early postoperative period. *Results*: The mean age of the study population was 64.8 ± 9.6 years; 59 patients (67.8%) were male, and 28 (32.2%) were female. The cut-off values for HALP, LCR, and albumin were 34.4, 0.61, and 38.55, respectively. Patients with low HALP scores had significantly shorter overall survival than those with high scores (15.8 vs. 19.3 months; *p* < 0.01). Similarly, patients with low LCR scores showed shorter survival than those with high scores (17.8 vs. 18.5 months; *p* < 0.01). High albumin levels were associated with significantly longer survival compared to low albumin levels (16.3 vs. 14.6 months; *p* < 0.01). *Conclusions*: Low HALP scores and low albumin levels were identified as significant independent prognostic factors for both disease-free and overall survival in patients with pancreatic adenocarcinoma.

## 1. Introduction

Pancreatic adenocarcinoma is among the most lethal and aggressive forms of cancer, with a one-year overall survival rate of 26% and a five-year survival rate of less than 5% [1]. According to the American Cancer Society’s 2022 data, pancreatic adenocarcinomas constitute 3% of all cancers but account for 7% of cancer-related deaths in the USA. Approximately 2000 new cases are diagnosed annually, with a similar incidence in males and females. The incidence increases with age. Despite advancements in early detection and treatment, the prognosis remains poor [2]. Due to the retroperitoneal location of the pancreas, pancreatic cancers are often diagnosed at advanced stages, contributing to high mortality and morbidity rates. High relapse rates further exacerbate these outcomes [3]. The etiology of pancreatic cancer involves both genetic and environmental risk factors, including gender, age, race, diabetes mellitus (DM), family history, genetic predisposition syndromes, and smoking.

There is a well-established relationship between the cancer development process and the systemic inflammatory response. The degree of systemic inflammatory response measured before surgery is associated with survival in patients with cancer. Whatever the cause behind systemic inflammation, it contributes to cancer development through effects such as increasing resistance to apoptosis around the tumor and stimulating angiogenesis [4,5]. With regard to this correlation, biochemical markers of inflammatory response, including albumin and C- reactive protein (CRP), have been proposed as prognostic indicators in solid cancers originating from various organs [6]. Hemoglobin, leukocyte and platelet counts are also known as factors that determine the prognosis of cancer patients [7,8]. Serum albumin is the most abundant blood plasma protein in humans and is produced by the liver. Albumin is also an important prognostic factor in oncological patients, and hypoalbuminemia has been shown indicate a poor prognosis [9]. Prognostic factors like the lymphocyte–CRP ratio (LCR) and hemoglobin, albumin, lymphocyte and platelet (HALP) score have been derived through combinations of these parameters that are obtainable through blood counts [10]. LCR is a ratio of lymphocytes and CRP levels and has been used as a prognostic factor in various cancers [11]. A low HALP score, which is the combination of hemoglobin, albumin, lymphocyte and platelet counts has been shown to be another sign of poor prognosis in cancer [11,12,13].

The aim of this study was to investigate whether the albumin value and the ratios of biochemical markers such as the hemoglobin, albumin, lymphocyte and platelet score (HALP), and the lymphocyte–C-reactive protein ratio (LCR) can predict survival and recurrence of the disease in patients with pancreatic adenocarcinoma. To our knowledge, there are fewer studies in the literature investigating the relationship between albumin, HALP score, LCR and survival and recurrence in pancreatic adenocarcinomas. Preoperative HALP and LCR values were calculated from laboratory data after a retrospective review of patients operated for pancreatic adenocarcinoma in our clinic. Furthermore, clinical follow-ups of these patients were conducted to access survival and recurrence data. Efforts were made to elucidate the relationships between calculated albumin, HALP, and LCR values derived from these data.

## 2. Materials and Methods

The study included 87 patients who underwent pancreaticoduodenectomy (Whipple procedure or total pancreatectomy) operations for pancreatic adenocarcinoma in the General Surgery Clinic of Ege University Hospital between January 2017 and December 2021. The data of the patients were obtained from the electronic medical records of the hospital and analyzed retrospectively. Ethics committee approval was received from Ege University Ethics Committee Commission (dated 23 March 2023 and Decision No: 23-3.1T/31). This study is registered with ClinicalTrials.gov, ID number NCT06553040.

Adult patients (over 18 years of age) who underwent a Whipple operation and total pancreatectomy due to pathologically confirmed pancreatic adenocarcinoma and had received adjuvant treatment were included in the study. Patients receiving neoadjuvant therapy, patients with incomplete clinicopathological and follow-up data, patients with other malignant tumors (distal choledochal, ampullary and duodenal cancers), and patients who died within 90 days of surgery were excluded from the study.

Patients were routinely followed until death due to disease relapse in accordance with a standardized protocol. Each patient was checked with clinical and laboratory examinations once a month or at longer intervals after surgery. If local or metastatic recurrence was suspected, imaging studies (computed tomography scans, magnetic resonance imaging, bone scans, or positron emission tomography/computed tomography) were recommended accordingly. Overall survival was defined as the time from surgery to death or last follow-up visit. Disease-free survival was defined as the time from surgery to tumor recurrence or last follow-up visit.

Inflammation markers such as HALP score and LCR value were obtained using pre-operative biochemical parameters.

**HALP score calculation**: Hemoglobin (g/dL) × Albumin (g/dL) × Lymphocyte (count/µL)/Platelet (count/µL).

**LCR calculation**: Lymphocytes (counts/μL)/CRP (mg/L).

### Statistical Analysis

The SPSS 24 statistical software package (Statistical Package for the Social Sciences—IBM^®^) was used to analyze the data collected in the study. Descriptive statistics regarding the distribution of responses to independent variables in the study are presented as numbers and percentages for categorical variables, and as means, standard deviations and medians for numerical variables. The compliance of continuous variables with the normal distribution assumption was evaluated with the Kolmogorov–Smirnow test. For pairwise and multiple comparisons, the Chi-square test and Fisher exact test were used for categorical variables, while the independent *t*-test, one-way ANOVA test or Kruskal–Wallis method was used for quantitative variables. The cut-off value with the minimum *p* value calculated from the log-rank χ^2^ test for overall survival was determined as the optimal cut-off value.

ROC (receiver operating characteristic) analysis was used to determine whether HALP and LCR values were prognostic indicators for clinical and pathological response prediction. The area under the ROC curves (AUC) values obtained as a result of ROC analysis were evaluated as follows: 0.9–1, excellent; 0.8–0.9, good; 0.7–0.8, moderate; 0.6–0.7, poor; and 0.5–0.6, unsuccessful. In the ROC analysis, the best cut-off point (maximum sensitivity and specificity) was determined. Sensitivity, specificity, positive-negative predictive values (PPV, NPV) and likelihood ratio (+) values were calculated to evaluate the success of the cut-off points determined after ROC analysis. The log-rank test was used to compare Kaplan–Meier survival curves. Log-rank tests were performed to compare patient survival between subgroups. Multivariable Cox regression survival analyses were performed to determine the independent association of all clinical features with survival. The results were evaluated at 95% confidence interval, and *p* < 0.05 was considered significant.

## 3. Results

### 3.1. Clinical Features and Survival and Mortality Characteristics of Patients

The clinical characteristics of the 87 patients are described in Table 1. The average age was 64.8 ± 9.6 (range, 41–109); 59 (67.8%) of the patients were male and 28 (32.2%) were female. The average survival time of the patients was 15.6 ± 10.6 months (range, 1–45), and the average disease-free survival time was 13.5± 15.5 (range, 0–72) months.

The average hemoglobin value of the patients was 12.8 ± 1.7 (range, 8.4–17.1); the average albumin value was 39.7 ± 5.3 (range, 26.4–51.0); the average lymphocyte value was 1914.7 ± 846.1 (µL) (range, 400–4780); the mean platelet value was 274,413.8 ± 87,668.7 (µL) (range, 124,000–614,000); and the mean CRP value was 18.8 ± 24.1 (mg/L) (range, 0.3–99.3).

While the average HALP score of the patients was 38.8 ± 22.3 (range, 5.9–132.2), their average LCR score was observed to be 0.66 ± 1.34 (range, 0.01–9.57).

The average number of metastatic lymph nodes in all patients was determined to be 22.3 ± 11.6 (range, 0–58). Mortality occurred in 72 (82.8%) patients during the follow-up period. One patient (1.1%) received radiotherapy plus chemotherapy.

Regarding the TNM classification of the tumors, it was observed that 15 (17.2%) were in the T1 stage, 45 (51.7%) were in the T2 stage, 17 (19.5%) were in the T3 stage, and 10 (11.5%) were in the T4 stage. Considering the N stages of the patients, it was observed that 20 (23.0%) were in the N0 stage, 31 (35.6%) were in the N1 stage, and 36 (41.4%) were in the N2 stage. Concerning the pathology of patients, it was observed that 12 (13.8%) had poor differentiation, 69 (79.3%) had moderate differentiation, and 6 (7%) had good differentiation. There was perineural invasion in 69 (79.3%) of the patients, lymphovascular invasion in 40 (46.0%), and local soft tissue invasion in 70 (80.5%) patients (Table 1).

### 3.2. Relationship Between Albumin, HALP Score and LCR Value and Clinical Features

**Figure 1:**
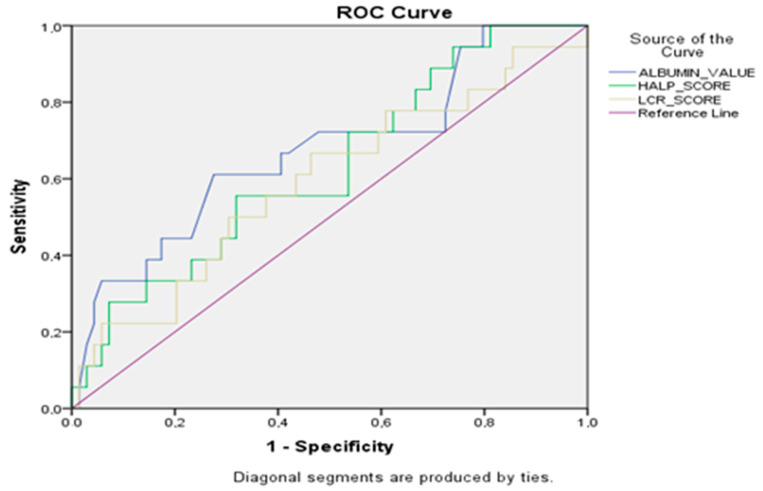
ROC analyses of albumin, HALP score and LCR score.

The sensitivity, selectivity, positive-negative predictive values and likelihood ratio (+) values calculated regarding the classification success of HALP values in response prediction according to the cut-off values determined as a result of ROC analysis of pancreatic adenocarcinoma patients are presented in Table 2 and Figure 1. For patients with pancreatic adenocarcinoma, the cut-off point for the albumin value was 38.55, the cut-off point for the HALP value was 34.4, and the cut-off point for the LCR value was 0.61. Regarding the classification success for these cut-off points, in patients with pancreatic adenocarcinoma, the sensitivity for the HALP score was 89.3% with a specificity of 55.6%, and the sensitivity for the LCR question was 79.8% with a specificity of 64.3%, while the sensitivity for the albumin value was 88.6% and the specificity was 78.8%.

The cut-off value of the HALP score was set as 34.4, providing the optimal classification for survival prediction. Low HALP scores were present in 42 patients. While the averages of age, disease-free survival, platelet and CRP levels of patients with low HALP scores were higher than in the high HALP group, their survival times, hemoglobin, albumin and lymphocyte values, and average lymph node numbers were lower than in the high HALP score group. The association between HALP scores and clinical features were as follows: survival (*p* = 0.048), disease-free survival (*p* = 0.032), Hb (*p* < 0.01), albumin (*p* < 0.01), lymphocyte (*p* < 0.001), and platelet (*p* = 0.006). It was observed that there were significant differences between CRP (*p* = 0.036) and resected lymph node (*p* = 0.027). In addition, a high HALP score was significantly associated with the patients being male, T1-2 stages, N1-2 stages, and receiving adjuvant chemotherapy (*p* < 0.05) (Table 3).

The cut-off value of LCR was set at 0.61, which provided the best classification for survival prediction. Low-level LCR was present in 70 patients. While the survival times, platelet, CRP levels and average number lymph nodes of patients with low LCR levels were higher than for the high LCR group, their average age, disease-free survival time, hemoglobin and albumin levels, and lymphocyte average numbers were observed to be lower than for the high LCR group. It was observed that there were significant differences between the average values of LCR and survival (*p* = 0.041), disease-free survival (*p* = 0.042), lymphocyte (*p* = 0.004), platelet (*p* = 0.008) and CRP levels (*p* < 0.001). In addition, the LCR score was significantly associated with patients being male, developing mortality, being in the T2-3 stages, being in the N1-2 stages, and receiving adjuvant chemotherapy (*p* < 0.05).

Low LCR levels were significantly associated with levels of perineural invasion, lymphovascular invasion, and surrounding soft tissue invasion (*p* < 0.05) (Table 4).

The cut-off value for albumin was set at 38.55, providing the best classification for survival prediction, and 35 patients had low levels of albumin. While the average age, platelet and CRP values of patients with low albumin values were higher than those of patients with high albumin values, it was observed that disease-free survival time, survival times, hemoglobin and lymphocyte levels and lymph node average numbers were lower than those of patients with high albumin values. It was determined that there were significant differences between the albumin value averages for survival (*p* = 0.043), disease-free survival (*p* = 0.017), Hb (*p* < 0.01), and lymphocyte count (*p* = 0.013). Moreover, low albumin value levels were significantly associated with perineural, perivascular and local soft tissue invasion levels (*p* < 0.05) (Table 5).

According to the Kaplan–Meier analysis, it was found that the overall survival time of patients with low HALP scores was significantly shorter (15.8 months) than patients with high HALP scores (19.3 months) (*p* < 0.01). In addition, it was found that the overall survival time of patients with low LCR scores was significantly shorter (17.8 months), and that of patients with high LCR scores was significantly longer (18.5 months) (*p* < 0.01). In addition, it was determined that the overall survival time of patients with low albumin values was significantly shorter (14.6 months) and that of patients with high albumin values was significantly longer (16.3 months) (*p* < 0.01).

According to the Kaplan–Meier analysis, it was found that the disease-free survival time (13.4 months) of patients with low HALP scores was significantly shorter than patients with high HALP scores (17.7 months) (*p* < 0.01). In addition, it was observed that the disease-free survival time of the patients (12.8 months) was significantly shorter than that of patients with high LCR scores (16.3 months) (*p* < 0.01). In addition, it was determined that the disease-free survival time (11.8 months) of patients with low albumin values was significantly shorter than patients with high albumin values (15.7 months) (*p* < 0.01).

It was observed that low HALP levels, low albumin values and high LCR levels were independent risk factors for early recurrence and short survival, regardless of gender, tumor stage and other variables.

In the univariate analysis, age, differentiation, tumor size, T stage, N stage, metastatic LN and lymphovascular invasion predicted survival (*p* < 0.05).

Univariate Cox regression analysis showed that albumin, LCR and HALP levels were all associated with median overall survival time. According to the multivariate Cox regression analysis, HALP score was shown to be an independent favorable factor for overall survival [HR = 2.367, 95% confidence interval (CI): 1.174–2.357, *p* < 0.001]. Additionally, LCR values were observed to be an independent positive factor for overall survival [HR = 1.188, 95% confidence interval (CI): 1.023–1617, *p* = 0.012]. Moreover, it was shown that albumin values were an independent favorable factor for overall survival [HR = 2.796, 95% confidence interval (CI): 1.878–4.286, *p* < 0.025] (Table 6).

## 4. Discussion

Numerous studies have explored prognostic factors for pancreatic adenocarcinoma, but no universally applicable marker for routine clinical practice has been identified yet. Many studies have shown that hemoglobin levels affect survival in patients with malignancies [14]. It is widely accepted that the systemic inflammatory response is associated with the prognosis of cancer patients [15,16]. Biomarkers derived from routine biochemical tests, such as hemoglobin, albumin, and CRP, are cost-effective and widely utilized across various diseases. Recent studies have identified a new marker, the HALP score, consisting of hemoglobin, albumin, lymphocytes, and platelets, reflecting both systemic inflammation and nutritional status. Previous studies have reported that it is associated with survival in patients with stomach [17], colorectal [18], kidney [19] and bladder cancer [20]. Models based on HALP score and LCR values have been shown to effectively identify patients with high survival risk in malignancies. However, there are very few studies investigating the relationship between HALP and survival after radical resection in patients with pancreatic cancer. Therefore, the aim of this study was to examine the correlation of albumin level, HALP scores and LCR values with survival and recurrence in patients with pancreatic adenocarcinoma.

Albumin is a negative acute-phase reactant synthesized in the liver. Hypoalbuminemia may result from malnutrition, hypercatabolism caused by cancer cells, and increased inflammation due to cytokine release, which plays a role in the survival of cancer patients. It is also widely used in various neoplastic diseases as a prognostic score for patients with sepsis. Serum albumin and hemoglobin levels, as measurable continuous variables, are some of the most commonly used indicators of a patient’s nutritional status and are also used to assess cancer progression and prognosis patients [7,12]. Decreases in serum albumin levels after surgery are an indicator of stress response. Therefore, reducing postoperative surgical stress may result in optimal early recovery of postoperative serum albumin levels. In fact, low albumin and hemoglobin levels are associated with poor survival of cancer patients [20,21,22]. In our study, the cut-off value of albumin for patients with pancreatic adenocarcinoma was determined as 38.55. It was found that the overall survival time of patients with low albumin value was significantly shorter (14.6 months) and that of patients with high albumin value was significantly longer (16.3 months) (*p* < 0.01). In addition, it was determined that the disease-free survival time of patients with low albumin value was significantly shorter (11.8 months) and that of patients with high albumin value was significantly longer (15.7 months) (*p* < 0.01).

Lymphocytes inhibit apoptosis by secreting TNF alpha and interferon gamma and contribute to long-term survival by preventing tumor migration and invasion. Lymphopenia is also frequently observed in advanced cancer patients and stimulates cancer progression [23,24]. Platelets play an important role in cancer formation and prognosis by increasing angiogenesis, migration and vascular permeability through growth factors. Metastasis is associated with platelet stimulation [25], and platelets protect cancer cells from immunological attack [26]. HALP is the integration of these four hematological parameters and has shown prognostic value for cancer patients [17].

The average survival time of all patients in our study was 15.6 ± 10.6 months (range, 1–45) and the average disease-free survival time was 13.5 ± 15.5 (range, 0–72) months. The general characteristics and median survival times of the patient population in our study were found to be compatible with those reported in the literature [27].

In our study, the cut-off value of HALP for patients with pancreatic adenocarcinoma was determined as 34.4, and the cut-off value for LCR was 0.61. In our study, it was determined that the overall survival time of patients with low HALP scores (15.8 months) was significantly shorter than that of patients with high HALP scores (19.3 months) (*p* < 0.01). In addition, another result we obtained from our study was that the disease-free survival time of patients with low HALP scores (13.4 months) was significantly shorter than that of patients with high HALP scores (17.7 months) (*p* < 0.01).

The median overall survival of the low-HALP was shorter than that of the high-HALP group, and this difference was statistically significant (*p* < 0.001). In this study, it was seen that a high HALP score was a good prognostic indicator, a result that was compatible with the literature [28,29,30].

There are many studies in the literature highlighting the prognostic importance of the HALP score. For example, a study involving 582 patients with resectable pancreatic cancer showed that a high preoperative HALP score was a prognostic factor for survival [31]. According to a study involving 355 patients diagnosed with esophageal squamous cell carcinoma, a high preoperative HALP score was identified as an independent and favorable prognostic indicator [32]. In a separate study by Topal et al., including 110 colorectal cancer patients who underwent surgery, it was observed that the prognostic significance of a low HALP score decreased in the presence of tumor budding [33]. Again, in a study in which 591 patients with locally confined gastrointestinal stromal tumors were followed after surgery, a low HALP score predicted short disease-free survival [34]. Ekinci et al. found in a study of 123 patients diagnosed with metastatic renal cell carcinoma that low HALP score was associated with poor prognosis [29]. In a study conducted on 204 patients with stomach cancer, they reported that a low HALP score numerically indicated a poor prognosis [28].

LCR, as a marker of inflammatory response, is safely used to predict disease-free survival and overall survival rates in patients diagnosed with colorectal cancer and gastric cancer. Low preoperative LCR values have been shown to be associated with worse overall survival and disease-free survival and with more advanced cancer stages [35,36].

The limitations of our study include its retrospective and single-center design, as well as its small patient population. However, the lack of a similar study comparing HALP and LCR inflammatory markers together for recurrence and survival in pancreatic adenocarcinoma patients increases the value of this study. Prospective studies with larger patient series are needed to confirm and standardize the data.

## 5. Conclusions

Preoperative HALP and LCR scores have been identified as effective predictors of postoperative overall survival and recurrence in patients with pancreatic adenocarcinoma. Notably, our findings suggest that overall survival can also be reliably predicted using albumin levels alone. These results highlight the potential clinical utility of incorporating inflammatory markers like HALP, LCR, and albumin into routine prognostic assessments.

However, to validate and generalize these findings, further research is needed. Large-scale, prospective, and well-designed studies are essential to confirm the prognostic significance of these markers and explore their integration with other diagnostic tools for improved clinical decision-making.

## Figures and Tables

**Table 1:** Clinicopathological characteristics of pancreatic adenocarcinoma patients, *n* (%).

Characteristic	*n* = 87
Age (year, mean ± std, min–max)	64.8 ± 9.6 (41–109)
Gender (male/female)	59 (67.8%)/28 (32.2%)
Survival (month, mean ± std, min–max)	15.6 ± 10.6 (1–45)
Disease-free survival (month, mean ± std, min–max)	13.5± 15.5 (0–72)
Hemoglobin (mean ± std, min–max)	12.8 ± 1.7 (8.4–17.1)
Albumin (mean ± std, min–max)	39.7 ± 5.3 (26.4–51.0)
Lymphocyte (mean ± std, min–max)	1914.7 ± 846.1 (400–4780)
Platelet (mean ± std, min–max)	274,413.8 ± 87,668.7 (124,000–614,000)
CRP (mean ± std, min–max)	18.8 ± 24.1 (0.3–99.3)
HALP score	38.8 ± 22.3 (5.9–132.2)
LCR value	0.66 ± 1.34 (0.01–9.57)
Resected lymph node (mean ± std, min–max)	22.3 ± 11.6 (0–58)
Mortality (yes/no)	72 (82.8%)/15 (17.2%)
T stage (1 stage/2stage/3 stage/4stage)	15 (17.2%)/45(51.7%)/17 (19.5%)/10 (11.5%)
N stage (0 stage/1stage/2 stage)	20 (23.0%)/31 (35.6%)/36 (41.4%)
TNM stage (1/2/3)	20 (22.9%)/50 (57.4%)/17 (19.5%)
Differentiation (Poor/Little/Moderately/Well)	1(1.1%)/12 (13.8%)/69(79.3%)/5(5.7%)
Perineural invasion (yes/no)	69 (79.3%)/18 (20.7%)
Lymphovascular invasion (yes/no)	40 (46.0%)/47(54.0%)
Local soft tissue invasion (yes/no)	70 (80.5%)/17(19.5%)

**Table 2:** Cut-off values and sensitivity results for albumin value, HALP score and LCR value of patients with pancreatic adenocarcinoma.

	With Pancreatic Adenocarcinoma-HALP	With Pancreatic Adenocarcinoma-LCR	With Pancreatic Adenocarcinoma-Albumin
AUC (95% CI)	0.655 (0.518–0.791)	0.771 (0.664–0.877)	0.826 (0.657–0.994)
*p* values	0.034	<0.001	0.028
Cut-off	34.41	0.61	38.55
Sensitivity (95% CI)	89.3% (63.5–98.5)	79.8% (62.2–97.9)	88.6% (69.7–99.3)
Specificity (95% CI)	55.6% (32.1–65.9)	64.3% (51–78.2)	78.8% (61.2–82.7)
PPV (95% CI)	26.8% (16.5–41.1)	37.5% (22.5–53)	34.2% (20.1–48.9)
NPV (95% CI)	94.9% (77.3–98.9)	96.7% (85.2–99.2)	95.4% (78.4–97.8)
LR+ (95% CI)	1.66 (1.18–2.14)	2.51 (1.59–3.32)	1.78% (1.21–2.85)

ROC: receiver operating characteristic, PPV: positive predictive value, NPV: negative predictive value, AUC: area under curve, CI: confidence interval, HALP: hemoglobin, albumin, lymphocyte, and platelet; LCR: lymphocytes and CRP. LR+: likelihoods ratio.

**Table 3:** Relationship between HALP scores and clinical features.

Variables	HALP		*p*
	Low (≤34.4, *n* = 42)	High (>34.4, *n* = 45)	
Age (year, mean ± std)	65.7 ± 8.1	63.9 ± 10.9	0.379
Survival (month, mean ± std)	15.8 ± 19.1	19.3 ± 11.1	0.048 *
Disease-free survival (month, mean ± std)	13.4 ± 9.0	17.7 ± 11.8	0.032 *
Hemoglobin (mean ± std)	12.3 ± 1.7	13.5 ± 1.3	<0.001 *
Albumin (mean ± std)	37.2 ± 5.3	42.0 ± 4.2	<0.001 *
Lymphocyte (mean ± std)	1456.0 ± 592.6	2342.9 ± 826.8	<0.001 *
Platelet (mean ± std)	300,714.3 ± 94,742.1	249,866 ± 73,359	0.006 *
CRP (mean ± std)	24.4 ± 25.0	13.6 ± 22.3	0.036 *
Lymph node (mean ± std)	19.1 ± 9.3	24.1 ± 13.2	0.027 *
Gender (male/female)	26/16	33/12	0.018 *
Mortality (yes/no)	36/6	36/9	0.338
T stage (1 stage/2 stage/3 stage/4 stage)	10/21/7/4	5/24/10/6	0.017 *
N stage (0/1/2)	12/17/13	8/14/23	0.046 *
Differentiation (poor/little/moderately/well)	0/5/33/4	1/7/36/1	0.367
Perineural invasion (yes/no)	34/8	35/10	0.461
Lymphovascular invasion (yes/no)	21/21	19/260,304	0.204
Local soft tissue invasion (yes/no)	33/9	37/8	0.436

One-way ANOVA test, Chi-square test, independent test, *p* < 0.05 significance, (*) means it is statistically significant.

**Table 4:** Relationships between LCR values and clinical features.

Variables	LCR		*p*
	Low (≤0.61, *n* = 70)	High (>0.61, *n* = 17)	
Age (year, mean ± std)	64.0 ± 7.9	68.9 ± 14.7	0.141
Survival (month, mean ± std)	17.8 ± 11.0	18.5 ± 8.6	0.041 *
Disease-free survival (month, mean ± std)	12.8 ± 12.8	16.3 ± 14.5	0.042 *
Hemoglobin (mean ± std)	12.7 ± 1.7	13.0 ± 1.4	0.437
Albumin (mean ± std)	37.3 ± 5.4	41.2 ± 4.7	0.181
Lymphocyte (mean ± std)	1788.6 ± 770.4	2434.1 ± 965.5	0.004 *
Platelet (mean ± std)	286,571.4 ± 88,428.7	224,352.9 ± 65,418.8	0.008 *
CRP (mean ± std)	23.0 ± 25.2	11.5 ± 0.8	<0.001 *
Resected lymph node (mean ± std)	22.4 ± 11.2	21.6 ± 13.4	0.654
Gender (male/female)	46/24	13/4	0.023 *
Mortality (yes/no)	58/12	14/3	0.012 *
T stage (1/2/3/4)	14/37/13/6	1/8/4/4	0.029 *
N stage (0/1/2)	15/22/33	5/9/3	0.031 *
Dıfferentıatıon (poor/little/moderately/well)	1/8/57/4	0/4/12/1	0.267
Perineural invasion (yes/no)	56/14	13/4	0.048 *
Lymphovascular invasion (yes/no)	33/37	7/10	0.038 *
Local soft tissue invasion (yes/no)	59/11	11/6	0.041 *

One-way ANOVA test, Chi-square test, independent test, *p* < 0.05 significance. (*) means it is statistically significant

**Table 5:** Relationship between albumin values and clinical features. (*) is statistically significant.

Variables	Albumin Value		*p*
	Low (≤38.55, *n* = 35)	High (>38.55, *n* = 52)	
Age (year, mean ± std)	65.2 ± 7.3	64.5 ± 11.0	0.748
Survival (month, mean ± std)	14.6 ± 9.1	16.3 ± 11.8	0.043 *
Disease-free survival (month, mean ± std)	10.0 ± 11.6	15.7 ± 17.3	0.017 *
Hemoglobin (mean ± std)	11.8 ± 1.7	13.4 ± 1.3	0.001 *
Lymphocyte (mean ± std)	1739.1 ± 722.2	2032.9 ± 907.8	0.013 *
Platelet (mean ± std)	277,400.0 ± 71,123.4	272,403.8 ± 97,866.1	0.796
CRP (mean ± std)	20.2 ± 21.3	17.9 ± 26.0	0.671
Resected lymph node (mean ± std)	21.1 ± 10.0	23.1 ± 12.6	0.446
Gender (male/female)	12/23	16/36	0.454
Mortality (yes/no)	4/31	11/41	0.188
T stage (1 stage/2stage/3 stage/4stage)	5/20/8/2	10/25/9/8	0.319
N stage (0 stage/1stage/2 stage)	7/13/15	13/18/21	0.862
Dıfferentıatıon (*poor/little/moderately/well*)	4/29/1/1	8/40/4/0	0.388
Perineural invasion (yes/no)	5/30	13/39	0.017 *
Lymphovascular invasion (yes/no)	18/17	29/23	0.042 *
Surrounding soft tissue invasion (yes/no)	5/30	12/40	0.023 *

**Table 6:** Univariate and multivariate Cox analyses for overall survival.

	Univariate Analysis		Multivariate Analysis	
Variable	HR (95%CI)	*p* Value	HR (95%CI)	*p* Value
Univariate analysis				
Age	1.361 (1.036–1.681)	0.012	-	-
Sex female vs. male	0.789 (0.562–1.265)	0.478	-	-
Adjuvant chemotherapy	1.221 (0.912–1.668)	0.23	1.067 (0.912–2.388)	0.231
Mortality	3.635 (2.321–5.684)	0.033	2.840 (0.915–4.671)	<0.001
Lymph node	1.856 (1.047–2.190)	0.047	1.719 (1.427–1.915)	<0.001
CRP	1.775 (1.365–2.215)	0.043	1.687 (1.380–2.165)	<0.001
Antrum	1.473 (1.175–1.876)	0.423	1.264 (1.135–1.387)	0.009
Differentiation	1.856 (1.241–2.732)	0.003	1.319 (0.982–1.862)	0.024
T Stage	2.894 (1.787–5.265)	0.014	2.478 (1.258–4.775)	0.037
N Stage	1.935 (1.125–2.092)	0.014	1.512 (1.157–1.913)	0.022
HALP score (≤34.4/>34.4)	2.635 (1.712–3.029)	<0.001	2.367 (1.174–2.357)	<0.001
LCR score (≤0.61/>0.61)	1.226 (1.068–1.681)	0.006	1.188 (1.023–1617)	0.012
Albumin (≤38.55/>38.55)	2.147 (1.536–2.489)	0.013	2.796 (1.878–4.286)	0.025
Positive retroperitoneal surgical margin	3.852 (1.836–7.910)	<0.001	3.589 (1.940–6.539)	<0.001
Local soft tissue invasion	2.284 (1.24–4.18)	0.008	3.412 (2.111–4.216)	<0.001
Lymphovascular invasion	2.367 (1.487–3.711)	<0.001	2.158 (1.517–2.943)	0.415
Perineural invasion	1.892 (0.987–2.822)	0.521	1.632 (1.365–2.573)	0.352

## Data Availability

The datasets used and analyzed during the current study are available from the corresponding author on reasonable request.
